# Role of Dynamic Actin Cytoskeleton Remodeling in Foxp3^+^ Regulatory T Cell Development and Function: Implications for Osteoclastogenesis

**DOI:** 10.3389/fimmu.2022.836646

**Published:** 2022-03-11

**Authors:** Sebastian Dohnke, Stephanie Moehser, Alexey Surnov, Thomas Kurth, Rolf Jessberger, Karsten Kretschmer, Annette I. Garbe

**Affiliations:** ^1^Osteoimmunology, Center for Regenerative Therapies Dresden (CRTD), Center for Molecular and Cellular Bioengineering (CMCB), Technische Universität Dresden, Dresden, Germany; ^2^Molecular and Cellular Immunology/Immune Regulation, Center for Regenerative Therapies Dresden (CRTD), Center for Molecular and Cellular Bioengineering (CMCB), Technische Universität Dresden, Dresden, Germany; ^3^Institute of Physiological Chemistry, Medical Faculty Carl Gustav Carus, Technische Universität Dresden, Dresden, Germany; ^4^Center for Molecular and Cellular Bioengineering, Technology Platform, Electron Microscopy and Histology Facility, Technische Universität Dresden, Dresden, Germany

**Keywords:** SWAP-70, Foxp3, actin dynamics, osteoclasts, Treg cell, T cell homeostasis

## Abstract

In T cells, processes such as migration and immunological synapse formation are accompanied by the dynamic reorganization of the actin cytoskeleton, which has been suggested to be mediated by regulators of RhoGTPases and by F-actin bundlers. SWAP-70 controls F-actin dynamics in various immune cells, but its role in T cell development and function has remained incompletely understood. CD4^+^ regulatory T (Treg) cells expressing the transcription factor Foxp3 employ diverse mechanisms to suppress innate and adaptive immunity, which is critical for maintaining immune homeostasis and self-tolerance. Here, we propose *Swap-70* as a novel member of the Foxp3-dependent canonical Treg cell signature. We show that *Swap-70^-/-^* mice have increased numbers of Foxp3^+^ Treg cells with an effector/memory-like phenotype that exhibit impaired suppressor function *in vitro*, but maintain overall immune homeostasis *in vivo*. Upon formation of an immunological synapse with antigen presenting cells *in vitro*, cytosolic SWAP-70 protein is selectively recruited to the interface in Treg cells. In this context, *Swap-70^-/-^* Treg cells fail to downregulate CD80/CD86 on osteoclast precursor cells by trans-endocytosis and to efficiently suppress osteoclastogenesis and osteoclast function. These data provide first evidence for a crucial role of SWAP-70 in Treg cell biology and further highlight the important non-immune function of Foxp3^+^ Treg cells in bone homeostasis mediated through direct SWAP-70-dependent mechanisms.

## Introduction

Foxp3^+^ regulatory T (Treg) cells are indispensable for the maintenance of immunological self-tolerance and homeostasis. While the majority of Treg cells in the periphery are thymus-derived (thymic Treg cells; tTreg cells), a proportion is generated extrathymically at peripheral sites (peripherally generated Treg cells; pTreg cells) (1–3). Treg cells are able to control immune responses by suppressing proliferation, activation, differentiation and effector function of various types of immune cells (4–7), whereby they protect the body against autoimmune diseases and excessive inflammation. In addition, there is increasing evidence that Treg cells exert key non-immune functions, including controlling metabolic and regenerative processes, the differentiation of hematopoietic stem cells, and the function of osteoclasts, bone resorbing cells and central mediators of skeletal diseases (8–11). Though it is now generally recognized that the interplay of bone and immune system has a crucial impact on health and disease, the exact function of Treg cells in bone homeostasis remains elusive.

Foxp3^+^ Treg cells are equipped with multiple mechanisms required to suppress target cells in a cell contact-dependent and/or -independent manner (12). While it has been suggested that Treg cells have the ability to suppress osteoclastogenesis *in vitro*, the mechanisms of suppression are still incompletely understood and controversially discussed: Some reports identified inhibitory cytokines as main players in Treg cell-mediated suppression of osteoclastogenesis (13–15), while other studies proposed cell-cell contact-dependent mechanisms (15–18). Since osteoclasts are derived from hematopoietic precursors, express a variety of immune receptors and are regulated similarly to dendritic cells (DCs) and macrophages, it has been proposed, that osteoclasts play a role in the active regulation of the immune system and can act for example as antigen presenting cells (APCs) (19–21). In this context, it was suggested that, similar to DCs, osteoclast progenitors express on their surface the costimulatory molecules CD80 and CD86, and that Treg cells can control osteoclastogenesis *via* engagement of CD80/CD86 by CTLA-4 (9, 22).

Appropriate Treg cell positioning in the tissue is the prerequisite for effective suppressive capacity *in vivo*. Migration and homing of Foxp3^+^ Treg cells to their anatomical sites of action, and the *in situ* retention of Treg cells upon immune synapse formation with tissue-resident APCs are driven by dynamic changes in the actin cytoskeleton (23, 24). SWAP-70 has been described as a unique F-actin regulatory protein, which binds and bundles F-actin (25), and has several non-redundant functions in various hematopoietic cells such as polarization (26), endocytosis (27), integrin-mediated adhesion (28, 29), migration (30), and homing (26). In many of these cell types, it was observed that SWAP-70 localizes to sites of active F-actin rearrangement (31–36). *Swap-70^-/-^* mice exhibit an osteopetrotic phenotype, characterized by increased bone mineral density caused by decreased resorptive activity of osteoclasts defective in F-actin ring formation (31, 35). In addition, earlier studies suggested that *Swap-70* might represent a previously unappreciated member of the Treg cell signature (37). This initial observation prompted us to hypothesize that the control of osteoclast differentiation and function by Treg cells could represent an additional, non-mutually exclusive mechanism underlying the dysregulated bone homeostasis in *Swap-70^-/-^* mice. Here, we report on the identification of functional defects in *SWAP-70-*deficient Foxp3^+^ Treg cells and their impact on T cell homeostasis and interaction with osteoclasts.

## Materials and Methods

### Mice

Foxp3^GFP^ mice (38) were crossed to *Swap-70*^-/-^ mice (39) to generate *Swap70^-/-^* x Foxp3^GFP^ mice. Foxp3^RFP/GFP^ mice expressing the Cre recombinase GFP (GFP-Cre) fusion protein as a Foxp3 BAC transgene and the RFP reporter from an internal ribosome entry site (IRES) downstream of the Foxp3 coding region (Foxp3^IRES-RFP^) have been previously described (40). Foxp3^GFP^ mice were bred to 2D2 mice (Jackson Laboratory), expressing the transgenic, self-reactive Vα3.2 (2D2) T cell receptor (TCR) specific for myelin oligodendrocyte glycoprotein [MOG, (41)] to generate Foxp3^GFP^ x 2D2-MOG mice. Foxp3^GFP^ x 2D2-MOG mice were then bred with *Swap70^-/-^* mice to obtain *Swap70^-/-^* x 2D2 x Foxp3^GFP^ mice. All mice were on C57/BL6 background and were housed and bred either at the animal facility of the CRTD, TU Dresden or the Experimental Center of the Medizinisch-Theoretisches Zentrum, TU Dresden. Animal experiments were performed as approved by the Regierungspräsidium Dresden (AZ 24-9168.24-1/2014-5, 24-9168.24-1/2014-1).

### Flow Cytometry and Cell Sorting

Single cell suspensions of thymus, spleen, mesenteric lymph nodes (mesLN) or pooled subcutaneous LN (scLN) (Lnn. mandibularis, Lnn. cervicales superficiales, Lnn. axillares et cubiti, Lnn. inguinales superficiales, and Lnn. subiliaci) were prepared using 70 µm cell strainers (BD Biosciences). DCs were isolated by enzymatic digestion of spleen slices using 0.2 mg/ml Dispase I (Roche), 0.2 mg/ml Collagenase D (Roche) and 25 µg/ml DNase (Qiagen) for 10 min at 37°C and labeled with biotinylated α-CD11c Abs, streptavidin-conjugated microbeads and fluorochromes for enrichment with the AutoMACS Pro magnetic cell separation system. Primary bone marrow-derived macrophages (BMMs) were isolated from long bones of C57BL/6 mice. Bones were rinsed with PBS containing 5% FCS (v/v) and 2 mM EDTA (Thermo Fisher, Invitrogen), followed by lysis of erythrocytes and filtration through a 100 µm mesh. Fluorochrome-conjugated antibodies against CD3 (145-2C11), CD4 (RM4-5), CD8 (53-6.7), CD11b (M1/70), CD11c (HL3), CD25 (PC61, 7D4), CD38 (90), CD44 (IM7), CD45.1 (A20), CD62L (MEL-14), CD69 (H1.2F3), CD80 (16-10-A1), CD86 (GL1), CD103 (M290), CD134/Ox40 (OX-46), cfms (AFS98), ICAM1 (eBioKAT-1), ICOS (7E.17G9), KLRG1 (2F1), Ly6c (HK1.4), Nrp1 (761705), TCRb (H57-597), CTLA-4 (UC10-4F10-11), Helios (22f6), SWAP-70 (polyclonal) were purchased from BD, eBiosciences or Novus Biologicals. For some experiments, cells were labeled with 5 µM eFluor670 cell proliferation dye (eBiosciences). Surface marker expression of CD62L and CD44 was employed to characterize naïve (CD62L^+^CD44^-^), effector-memory (CD62L^-^CD44^+^) and central memory (CD62L^+^CD44^hi^) CD8^+^ T cell subsets, as well as naïve (CD62L^+^CD44^-^) and memory-type (CD62L^-^CD44^+^) CD4^+^ Tcon/Treg cell subsets.

Biotin-conjugated mAbs were additionally labeled with Streptavidin-PE-Cy7 or Streptavidin-ef450 (BD Biosciences, eBiociences). The polyclonal rabbit anti-SWAP-70 antibody (produced in our lab or purchased from Novus Biologicals) was conjugated with α-chain IgG-AF647 (Thermo Fischer, Invitrogen). Prior to cell sorting, cells were enriched for CD4^+^ or CD25^+^ cells using biotinylated mAbs directed against CD4 or CD25, respectively, streptavidin-conjugated microbeads + fluorochromes and the AutoMACS Pro magnetic cell separation system (Miltenyi Biotec). Where indicated, CD3-, CD4- and CD8-depleted splenocytes were used as APCs. For flow cytometric analysis, samples were analyzed on a LSRII or LSR Fortessa. Cell sorting was performed using a FACS Aria II, FACS Aria III or FACS Aria Fusion system (all BD). Cell-cell interactions and/or subcellular localization of Ags detected by fluorochrome-labeled Abs were analyzed by imaging flow cytometry (Amnis Imagestream X Mark II).

### T Cell Culture

T cells were cultured in RPMI complete, consisting of RPMI 1680 medium supplemented with 1 mM Sodium pyruvate, 1 mM HEPES, 2 mM Glutamax, 100 U/ml Penicillin, 100 µg/ml Streptomycin, 100 µg/ml Gentamycin, 0.1 mM non-essential amino-acids, 0.55 mM β-mercaptoethanol and 10% FCS (v/v) (all Thermo Fisher, Life Technologies). If not stated otherwise, T cells were cultured in 96-well round-bottom plates (Greiner) at 37°C and 5% CO_2_ (Hera Cell 240, Thermo Fisher Scientific) in 200 µl RPMI complete medium. For short-term stimulation, *ex vivo* sorted cells were cultivated for 4 h in the presence of 50 ng/ml phorbol 12-myristate 13-acetate (PMA, Sigma Aldrich) and 200 ng/ml ionomycin (Iono, Calbiochem). Where indicated, 2 μM cyclosporine A (CsA, Sigma Aldrich) was added 1 h prior to stimulation. For polyclonal TCR stimulation, *ex vivo* FACS-purified T cells were cultured in RPMI complete in the presence of Mouse T-Activator CD3/CD28 Dynabeads (Gibco, Thermo Fisher Scientific). For some experiments, 100-1000 U/ml human recombinant IL-2 (Teceleukin, Roche) was added, as indicated. Ag-specific stimulation of 2D2 receptor expressing T cells was performed in the presence of purified DCs (DC: Treg:Tcon cell ratio 1:5:5) and 10 µg/ml MOG_35-55_ peptide (H-Met-Glu-Val-Gly-Trp-Tyr-Arg-Ser-Pro-Phe-Ser-Arg-Val-Val-His-Leu-Tyr-Arg-Asn-Gly-Lys-OH, JPT Peptide Technologies). T cells were cultured for 48 h in 96-well round-bottom plates at 37°C and 5% CO_2_ in 200 µl RPMI complete medium.

### *In Vitro* Suppression Assay

The suppressive capacity of Foxp3^+^ Treg cells was assessed in co-culture with T responder (Tresp) cells. To this end, CD4^+^Foxp3^GFP-^CD25^-^CD44^-^CD62L^+^ Tresp cells were FACS-purified and labeled with eFluor670 proliferation dye. CD4^+^Foxp3^GFP+^CD25^+/-^ Treg cells were FACS-purified from CD4 bead-enriched LNs (mesLN, scLN) and spleen. 5 x 10^4^ Tresp cells were co-cultured with 2.5 x 10^5^ irradiated APCs and Treg cells in different ratios and in the presence of 1 µg/ml soluble α-CD3ϵ. Tresp cell proliferation and CD25 expression was assessed at indicated time points by flow cytometry.

### Co-Cultivation of Foxp3^+^ Treg Cells and Osteoclasts

FACS-purified CD4^+^CD25^+^Foxp3^GFP+^ Treg cells were TCR-stimulated for 24 h in the presence of 2 α-CD3/CD28-coated beads/cell and 1000 U/ml IL-2 (Teceleukin, Roche) in RPMI complete. BMMs were seeded in minimal essential α-Medium (α-Mem, Merck, Biochrom) supplemented with 20% FCS, 100 U/ml penicillin, 100 µg/ml streptomycin, 2 mM glutamine (all Thermo Fisher, Life Technologies) and 25 ng/ml M-CSF 12 h before activated Treg cells and freshly isolated 20 Gy irradiated anti-CD3/CD4/CD8-depleted splenocytes were transferred to the BMM culture. At day 0, osteoclast differentiation medium (α-Mem with 20% FCS, 100 U/ml penicillin, 100 µg/ml streptomycin, 25 ng/ml M-CSF, 40 ng/ml RANKL) and 5 µg/ml α-CD3ϵ was added. The osteoclast differentiation medium was exchanged at day 2 and 5 and supplemented with fresh α-CD3ϵ. M-CSF was obtained from sterile filtrated L929-M-CSF-conditioned medium (42), human soluble RANKL was produced by recombinant expression in *Pichia pastoris*, kindly provided by B. Hoflack (Biotechnology Center Biotec, Technische Universität Dresden). Cells were stained for tartrate-resistant acid phosphatase (Acid phosphatase, Leukocyte kit, Merck, Sigma Aldrich) at day 6-7 according to the manufacturer’s protocol, imaged by light microscopy (Apotome inverse, Zeiss) and analyzed with Cell Profiler V.3.0. For some experiments, the cells were harvested at indicated time points and analyzed by flow cytometry or imaging flow cytometry (Amnis Imagestream X Mark II), respectively.

### CTX Elisa

Pre-osteoclast-Treg cell co-culture was seeded on bovine bone slices (#1BON1000, Nordic Bioscience) at different ratios (Treg cell: monocyte ratio: 1:100, 1:10, 1:5; control: without Treg cells). The cell culture supernatants were harvested on day five of the co-culture and carboxy-terminal collagen crosslinks (CTX) concentrations were determined from one sample of each titration step by (CTX-I) ELISA (IDS) and Tecan infinite 200Pro (Tecan i-control V 1.10.4.0 Software) according to the manufacturer’s recommendations. Samples were diluted according to the protocol.

### Scanning Electron Microscopy

The bone slices were harvested at day 7, fixed in 1% glutaraldehyde in PBS followed by dehydration in a graded ethanol series and critical point drying using the Leica CPD300 drier. Dried samples were mounted on 12 mm aluminium stubs using conductive carbon pads as a substrate and sputter coated with gold (Baltec SCD 050). The samples were analyzed by a table top scanning electron microscope (Hitachi TM 1000) running at 15kV acceleration voltage and using a backscatter electron detector.

### Gene Expression Analysis

Total RNA was extracted from 1-5 x 10^5^ FACS-purified cells from pooled scLN, mesLN and spleen with the RNeasy Micro kit (all Qiagen) according to the manufacturer’s protocol. For quantitative RT-PCR, cDNA was synthesized according to the manufacturer’s recommendations (SuperScript II reverse transcriptase and Oligo-d(T) primers from Invitrogen). The expression levels of mRNA transcripts were determined using the QuantiTect SYBR Green PCR Kit (Qiagen), a Mastercycler ep realplex thermal cycler (Eppendorf) and the following primers: *Cd4:* 5’-CTC ACA GGT CAA AGT ATT GTT G-3’; 5’-GAG AGT CAG CGG AGT TCT C-3’; *CD25:* 5’-ACC ACA GAC TTC CCA CAA CCC ACA-3’; 5’-CGC TCA GGA GGA GGA TGC TGA TGA-3’; *Foxp3* 5’-CCC AGG AAA GAC AGC AAC CTT-3’; 5’-CAA ACA GGC CGC CGT CTG GAG CC-3’; *Il-2*: 5’-CCT GAG CAG GAT GGA GAA TTA CA-3’; 5’-TCC AGA ACA TGC CGC AGA G-3’; *Swap-70*: 5’-ATG TGA GCG AGG ATC TGA AAG-3’; 5’-ATG GTG GAG TAA ATG GCC TG-3’. Expression levels were normalized to *Gapdh* or *HPRT*, as indicated (*Gapdh*: 5’-TGT GAT GGG TGT GAA CCA CGA GAA -3’; 5’-GAG CCC TTC CAC AAT GCC AAA GTT-3’; *HPRT*: 5’-GTC AAC GGG GGA CAT AAA AG-3’; 5’-AGG GCA TAT CCA ACA ACA AAC-3’).

### Statistical Analysis

Statistical significance was assessed using Prism 6.07. For statistical analysis of two groups, student’s t-test was applied. Differences were considered as significant when: * P ≤ 0.05; ** P ≤ 0.01; *** P ≤ 0.001; or **** P ≤ 0.0001.

## Results

### *Swap-70* Is Constitutively Expressed in Foxp3^+^ Treg Cells and Up-Regulated Upon TCR-Mediated Stimulation in Initially Naïve Swap-70^-^ CD4^+^ Tcon

**Figure 1:**
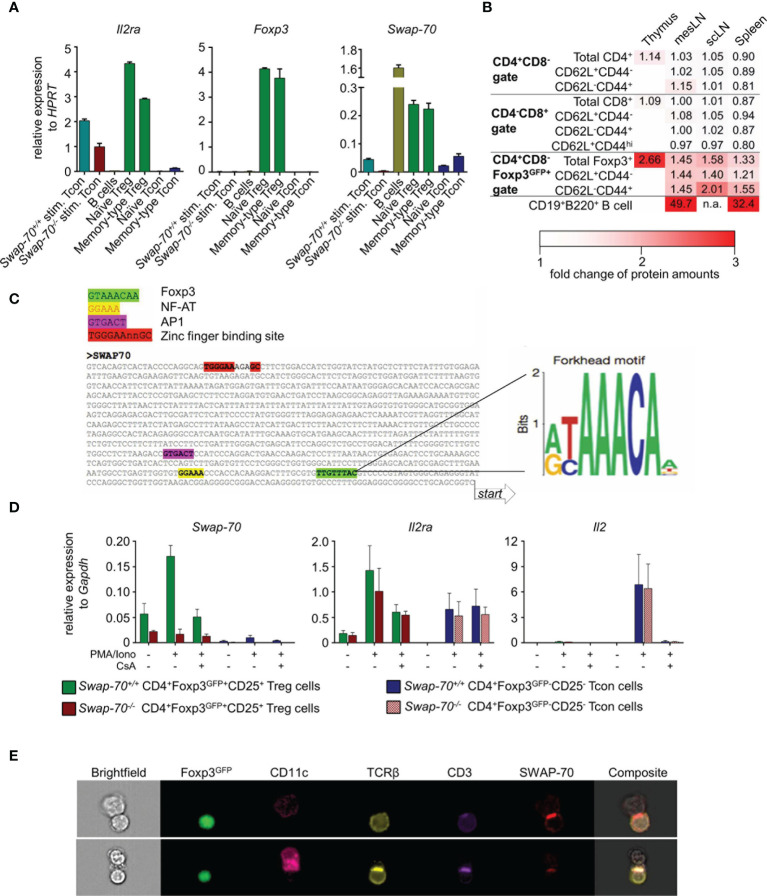
Constitutive SWAP-70 expression in Foxp3^+^ Treg cells and inducible *Swap-70* expression in short-term stimulated and memory-type CD4^+^ Tcon cells. **(A)** mRNA expression levels of *IL2ra, Foxp3* and *Swap-70* determined by real-time RT-PCR employing FACS-purified T cell populations (naïve Treg cells, memory-type Treg cells, naïve Tcon cells and memory-type Tcon cells). Where indicated, naïve *Swap-70^+/+^* and *Swap-70^-/-^* Tcon were short-term stimulated *in vitro* in the presence of PMA/Ionomycin. Peripheral B cells were used as positive control. Mean values ± S.D. of relative expression determined in a representative experiment (from three independent experiments) are shown for indicated genes. **(B)** Flow cytometric protein level analysis of intracellular (IC) SWAP-70 expression in electronically gated thymic and peripheral (scLN, mesLN, and spleen) T cell subsets. B cells were used as positive control. MFIs of samples from 6 individual mice (n=1 for CD19^+^B220^+^ B cells) were normalized to *Swap-70^-/-^* controls (set to 1). Data are shown as heatmap displaying *Swap-70^+/+^* to *Swap-70^-/-^* fold-change values of SWAP-70 expression. See also **Supplementary Figure S1**. **(C)** Identification of putative Foxp3, NF-AT, AP1 and zinc finger binding sites in the promoter region of *Swap-70* by multiple sequence alignment (left). **(D)** mRNA expression levels of *Swap-70, IL2ra* and *Il2* of Treg cells (green, red) and Tcon (blue, purple) cells FACS-purified from *Swap-70^+/+^* (green, blue) and *Swap-70^-/-^* mice (red, purple) were cultured for 1h in the absence or presence of cyclosporine A (CsA) prior to a 4h stimulation with PMA/Iono as indicated. Mean values ± S.D. of relative expression (n=4 from 2 independent experiments), determined in triplicate, are shown for indicated genes. **(E)** Representative imaging flow cytometry of co-cultures of FACS-purified Treg cells and Tcon cells expressing the transgenic 2D2 MOG-specific TCR cultured for 48h in the presence of CD11c^+^ dendritic cells (DCs) (DC: Treg:Tcon cell ratio 1:5:5) and 10 µg/ml MOG peptide. Cell clusters were analyzed for Foxp3^GFP^, CD11c, TCRβ, CD3 and IC SWAP-70 expression. DC-Treg cell interactions between *Swap-70^+/+^* DCs and *Swap-70^+/+^* Treg cells are shown in both rows.

Consistent with a previous study (37), qPCR analysis of FACS-purified T and B cell populations from pooled peripheral lymphoid organs revealed that *Swap-70* expression in truly naïve CD4^+^ T cells (CD25^-^Foxp3^GFP-^CD62L^+^CD44^-^) was at the limit of detection, but significantly increases upon short-term stimulation with PMA/Ionomycin *in vitro*. Notably, *Swap-70* expression was comparable in short-term stimulated and antigen-experienced memory-type CD4^+^ T cells (CD25^-^Foxp3GFP^-^CD62L^-^CD44^+^) (**Figure 1A**, right). As expected, *Il2ra* mRNA was constitutively expressed in naïve and memory-type Treg cells and up-regulated in CD4^+^ Tcon upon short-term stimulation, while *Foxp3* expression was restricted to Treg cells (**Figure 1A** left, middle). *Swap-70* mRNA was constitutively expressed in naïve and memory-type Foxp3^+^ Treg cells, with similar expression levels. By comparison, *Swap-70* expression in short-term stimulated Tcon cells accounted for about 24% of the levels detected in Foxp3^+^ Treg cells. Due to the known high-level constitutive expression of SWAP-70 in the B cell lineage (43), CD19^+^B220^+^ B lineage cells were included for comparison. Notably, expression levels of *Swap-70* mRNA in peripheral Foxp3^+^ Treg cells corresponded to approximately 15% of that of CD19^+^B220^+^ B lineage cells (**Figure 1A**, right).

T cell development and Foxp3^+^ tTreg commitment occurs in the thymus. Taking advantage of Foxp3^GFP^ reporter mice harboring a GFP-Foxp3 fusion protein reporter knock-in allele allowed us to track Foxp3^+^ thymocytes which are committed to the tTreg cell lineage by flow cytometry (38).

Intracellular (IC) SWAP-70 staining revealed that in the thymus, SWAP-70 protein was highly expressed in Foxp3^GFP+^ CD4 single positive (SP) thymocytes, while its expression was not detectable in Foxp3^GFP-^ CD4SP or CD8SP thymocytes (**Figure 1B** and **Supplementary Figure S1**). In peripheral lymphoid tissues, SWAP-70 protein was neither detectable in naïve, effector-memory and central memory CD8^+^.

T cells, nor in naïve and memory-type CD4^+^ Tcon cells, but was exclusively expressed in CD4^+^Foxp3^+^ Treg cells. Within the Foxp3^+^ Treg cell population, memory-type Treg cells exhibited the highest levels of SWAP-70 expression (**Figure 1B** and **Supplementary Figure S1**). While Foxp3^GFP^ expression was readily detectable in both, a fraction of CD4SP thymocytes and of peripheral CD4^+^ T cells, the two anatomical sites clearly differed in the level of SWAP-70 expression: based on the MFI values of anti-SWAP-70 Ab fluorescence, expression levels of SWAP-70 protein in CD4^+^Foxp3^+^ thymocytes were approximately twice as high as in the total CD4^+^Foxp3^+^ Treg cell population in the periphery. Normalized MFI values showed that expression levels of SWAP-70 protein in intra- and extrathymic Foxp3^+^ Treg lineage cells accounted for 4-7% of that of CD19^+^B220^+^ B lineage cells (**Figure 1B**).

### *Swap-70* mRNA Expression Is Controlled by the Nuclear Factor of Activated T cells (NF-AT)

Next, we sought to gain insight into the transcriptional regulation of *Swap-70* in T cells and employed a bioinformatics approach to predict T cell-relevant TF binding sites. Importantly, the Forkhead motif (37) could be identified in close proximity (108 bp), upstream of the transcription start site, implying that *Swap-70* is under the direct control of Foxp3. In addition, the NF-AT consensus sequence and a putative binding site for AP-1 were located 135 bp and 292 bp upstream of the transcription start site, respectively. Furthermore, a zinc finger binding motif, which is a putative binding site for TFs of the zinc finger superfamily, such as Ikaros proteins, was located 974 bp upstream from the transcription start site of *Swap-70* (**Figure 1C**).

To determine whether *Swap-70* is directly regulated by NF-AT, we assessed the impact of cyclosporine A (CsA) treatment on the up-regulation of *Swap-70* in PMA/Ionomycin-stimulated, initially naive CD4^+^ Tcon and Foxp3^+^ Treg cells. Our data revealed that PMA/Ionomycin stimulation increased *Swap-70* mRNA levels (3-fold compared to unstimulated) while pretreatment with CsA prevented this effect (**Figure 1D**, left). This response to PMA/Ionomycin stimulation ± CsA is reminiscent of other direct Foxp3 target genes of the Treg signature, such as *Il2ra* [**Figure 1D**, middle, (37, 44)]. In addition, we observed that *Swap-70^-/-^* Treg cells up-regulated *Il2ra* mRNA upon PMA/Iono stimulation comparable to their *Swap-70^+/+^* counterparts and that CsA completely abrogates *Il2* mRNA transcription in *Swap-70^+/+^* and *Swap-70^-/-^* CD4^+^ Tcon cells (**Figure 1D**, right). In summary, our results identify *Swap-70* as a direct Foxp3/NFAT target and a novel member of the canonical Treg cell signature.

### SWAP-70 Is Recruited to the Immunological Synapse of Activated Treg Cells

To address whether SWAP-70 is involved in cytoskeletal rearrangement at the immunological synapse formed between CD4^+^ Treg/Tcon cells and DCs, we took advantage of 2D2 MOG-specific TCR transgenic Foxp3^GFP^ mice. FACS-purified Foxp3^+^ Treg cells and CD4^+^ Tcon cells expressing the transgenic 2D2 TCR were co-cultured in the presence of DCs and MOG peptide. Imaging flow cytometry at day 2 of co-culture revealed that Treg cells could be reliably identified by the expression of TCRβ, CD3 and Foxp3^GFP^ and DCs by CD11c. SWAP-70 was detected in stimulated Foxp3^+^ Treg cells and DCs, but was barely detectable in stimulated CD4^+^ Teff cells (**Figure 1E** and data not shown), which is consistent with our qPCR data (**Figure 1A**). Analysis of DC-T cell interactions revealed clustered expression of TCRβ and CD3 at the T cell-APC interface, indicating the formation of the central supramolecular activation cluster (cSMAC) of the immunological synapse. In SWAP-70-proficient Treg cells, SWAP-70 was recruited to the immunological synapse and co-localizes with the cluster. These data provide first direct evidence that, upon TCR : MHC/peptide engagement, cytosolic SWAP-70 protein is selectively recruited to the immunological synapse in primary T cells.

### Expression of SWAP-70 During the Instructive Phase of tTreg Cell Development

**Figure 2:**
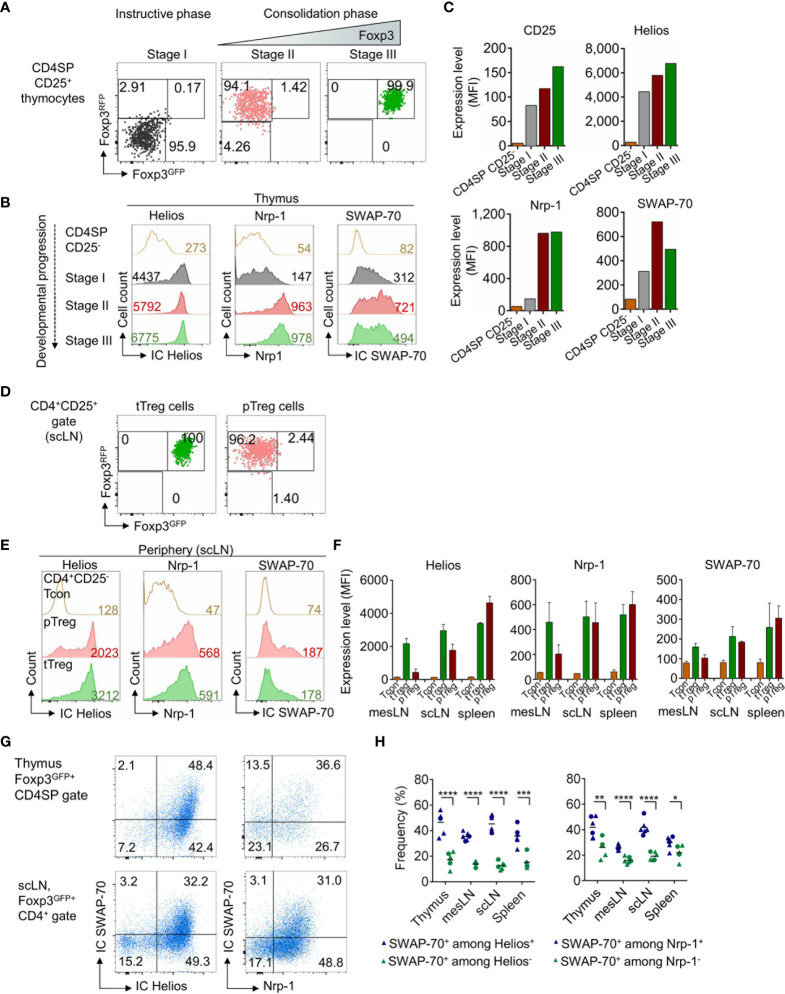
SWAP-70, Helios and Nrp-1 expression during tTreg cell development and in extrathymic Treg cells. **(A)** Postsort analysis of FACS-purified CD4SP CD25^+^ Treg precursor stages from the thymus of Foxp3^RFP/GFP^ mice showing Foxp3^GFP^ and Foxp3^RFP^ expression. **(B)** IC Helios, Nrp-1 and IC SWAP-70 expression during the instructive phase (Foxp3^RFP-^Foxp3^GFP-^CD25^+^ CD4SP, stage I, grey) and both stages of the consolidation phase (Foxp3^RFP+^Foxp3^GFP-^CD25^+^ CD4SP, stage II, red and Foxp3^RFP+^Foxp3^GFP+^CD25^hi^ CD4SP, stage III, green) of tTreg cell lineage commitment. **(C)** Bar charts depict representative expression levels (MFI) of CD25, Helios, Nrp-1 and SWAP-70 in CD4SP CD25^-^ thymocytes and during Treg cell development (stages I-III). **(D)** Postsort analysis of FACS-purifed tTreg (Foxp3^RFP+^Foxp3^GFP+^CD25^+^CD4^+^) and pTreg cells (Foxp3^RFP+^Foxp3^GFP-^CD25^+^CD4^+^) cells isolated from scLN of Foxp3^RFP/GFP^ mice showing Foxp3^GFP^ and Foxp3^RFP^ expression. **(E)** IC Helios, Nrp-1 and IC SWAP-70 expression in CD4^+^CD25^-^ Tcon, pTreg and tTreg cells. **(F)** Bar charts depict mean expression levels (MFI) of Helios, Nrp-1 and SWAP-70 in CD4^+^CD25^-^ Tcon, pTreg and tTreg cells isolated from different anatomical locations (mesLN, scLN, and spleen). **(G)** Representative flow cytometry of electronically gated Foxp3^GFP+^CD4SP thymocytes (top) and extrathymic Foxp3^GFP+^CD4^+^ Treg cells (scLN, bottom) from Foxp3^GFP^ mice stained for IC SWAP-70 in combination with IC Helios or Nrp-1. **(H)** Graph illustrates frequencies of IC SWAP-70 expressing cells among the respective Foxp3^GFP+^CD4^+^CD8^-^ cell subsets. Symbols and horizontal lines indicate individual mice and mean values, respectively (blue symbols: SWAP-70^+^ among Helios^+^ or Nrp-1^+^ cells, green symbols: SWAP-70^+^ among Helios^-^ or Nrp-1^-^ cells) mean of 6 individual mice shown, circles: 8-9-week-old, triangles 10-13-week-old mice. Numbers in dot plots indicate representative frequencies of gated cells within the respective gate. Numbers in histograms indicate representative MFIs of gated cells. See also See also **Supplementary Figure S2**.

Considering that Foxp3 expression is not a prerequisite of Treg cell lineage commitment, but rather a consequence thereof (45, 46), we next examined SWAP-70 expression in thymocytes that are still lacking Foxp3 protein expression but are already committed to the tTreg cell lineage. To this end, we employed Foxp3^RFP/GFP^ mice (40), which allow to track developmental stages of thymic Treg cell lineage development based on differential GFP and RFP expression. Early tTreg cell precursors in the instructive phase (stage I) exhibit a Foxp3^RFP-^Foxp3^GFP-^CD25^+^ CD4SP phenotype, Foxp3^RFP+^Foxp3^GFP-^CD25^+^ CD4SP tTreg cells represent precursors in the early consolidation phase (stage II), while Foxp3^+^ thymocytes at a later developmental stage are Foxp3^RFP+^Foxp3^GFP+^CD25^hi^ CD4SP (stage III) (47, 48). Following FACS-purification of CD25^+^ CD4SP Treg precursor stages according to the differential expression of RFP and GFP (**Figure 2A** and **Supplementary Figure S2A**), cells were stained for expression of IC SWAP-70 and co-expression of IC Helios and Nrp-1 (**Figure 2B** and **Supplementary Figure S2B**). Our analyses revealed high expression levels of Helios, but low expression levels of CD25, Nrp-1 and SWAP-70 during the instructive phase of Treg cell development (stage I). Notably, CD25, Nrp-1 and SWAP-70 were up-regulated in the early consolidation phase (stage II), concomitant with Foxp3^IRES-RFP^, and continuously expressed during the late consolidation phase (stage III), accompanied by Foxp3^Cre-GFP^. MFI values indicated a gradual upregulation of Helios and CD25 during the early and late consolidation phase of tTreg cell lineage commitment (**Figures 2B, C**). Overall, these data show that SWAP-70 expression is not yet detectable in CD25^-^ CD4SP thymocytes, but is induced concomitantly with CD25, Helios and Nrp-1 in the instructive phase of tTreg cell lineage commitment (stage I) and is progressively up-regulated to high levels during developmental progression and lineage consolidation (stage II and III). The observed kinetics suggests a mechanism of TCR-mediated induction and subsequent Foxp3-mediated amplification/stabilization of SWAP-70 expression during tTreg cell development.

### SWAP-70 Expression in Mature Treg Cells Correlates With Expression of CD44, Helios and Nrp-1

There is growing evidence that Helios and Nrp-1 expression is not unequivocally restricted to tTreg cells and that both molecules can be up-regulated upon TCR stimulation in pTreg cells (40, 47, 49, 50). Since our data revealed a correlation between SWAP-70 expression and Helios and Nrp-1 in tTreg cells, we next assessed whether SWAP-70 expression is restricted to the tTreg lineage. For this aim, tTreg and pTreg cells were FACS-purified from scLN of Foxp3^RFP/GFP^ mice (**Figure 2D** and **Supplementary Figure S2C**) and stained for expression of IC SWAP-70, IC Helios and Nrp-1 (**Figure 2E** and **Supplementary Figure S2D**). Similar to Helios and Nrp-1, SWAP-70 expression could not only be detected in tTreg but also to a similar extend in pTreg cells and can therefore not be considered as a tTreg lineage specific marker (**Figures 2E, F**). Consistent with previous reports (49, 51), the vast majority of Foxp3^+^ CD4SP thymocytes co-expressed high levels of Helios (> 90%), whereas in the periphery Helios expression was restricted to a subset of CD4^+^Foxp3^+^ Treg cells (**Figures 2E, F**). Importantly, our data clearly show that SWAP-70 expression correlates with Helios and Nrp-1 expression both in thymus and peripheral lymphoid tissues: high expression levels of SWAP-70 coincided with high Helios and Nrp-1 expression, while CD4^+^Foxp3^+^ Treg cells with a Helios^low^Nrp-1^low^ phenotype were almost negative for SWAP-70 (**Figures 2G, H****)**. In summary, these results reveal a distinct expression pattern of SWAP-70 within the Foxp3^+^ Treg cell population and suggest SWAP-70 as a putative marker for Helios^hi^Nrp-1^hi^ Treg cells.

### The Role of SWAP-70 in Immune Homeostasis

**Figure 3:**
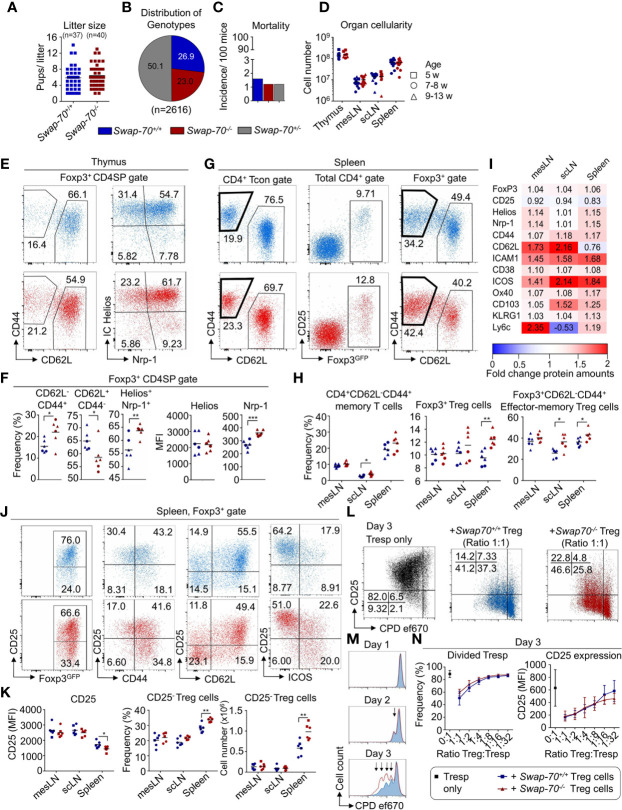
Impact of SWAP-70 on immune homeostasis. **(A–D)** *Swap-70* deficiency does not affect fertility, mortality or organ cellularity. **(A)** Number of litters and litter sizes of 8 *Swap-70^+/+^* (blue) and 6 *Swap-70^-/-^* (red) mating trios (2♀ x 1♂) within a period of 15 months. Symbols and horizontal lines indicate individual mice and mean values, respectively. **(B)** Distribution of genotypes and **(C)** mortality rate of live born *Swap-70* x Foxp3^GFP^ pups. Pie and bar chart display data from 2616 mice monitored over a period of 36 months (*Swap-70^+/+^*: blue; *Swap-70^-/-^*: red; *Swap-70^+/-^*: grey). **(D)** Total organ cellularity of *Swap-70^+/+^* and *Swap-70^-/-^* mice as indicator for multi-organ autoimmunity. Symbols and horizontal lines indicate individual mice and mean values, respectively (squares: 5-week-old, circles: 7-8-week-old; triangles: 9-13-week-old mice, n= 12 per genotype). **(E)** Representative flow cytometry of CD44, CD62L, IC Helios and Nrp-1 expression of electronically gated Foxp3^GFP+^ CD4SP thymocytes from *Swap-70^+/+^* (blue) and *Swap-70^-/-^* (red) Foxp3^GFP^ mice. **(F)** Graphs depict frequencies of the respective cell populations among electronically gated Foxp3^GFP+^ CD4SP thymocytes and Helios/Nrp-1 protein expression levels (MFI) of electronically gated Foxp3^GFP+^ CD4SP thymocytes. **(G)** Representative flow cytometry of CD44 and CD62L expression among gated CD4^+^ Foxp3^GFP-^CD25^-^ Tcon, Foxp3^GFP^ and CD25 expression among total CD4^+^ and CD44 and CD62L expression among CD4^+^Foxp3^GFP+^ Treg cells in the spleen of *Swap-70^+/+^* (blue) and *Swap-70^-/-^* (red) mice. **(H)** Graphs illustrate frequencies of memory T cells, Treg cells and effector-memory Treg cells in mesLN, scLN, and spleen. **(I)** Normalized protein expression levels in *Swap-70^-/-^* Foxp3^GFP+^ Treg cells from mesLN, scLN and spleen determined by surface and/or intracellular flow cytometry staining of the indicated proteins. MFIs of 4-6 individual mice were normalized to *Swap-70^+/+^* Foxp3^GFP+^ controls (set to 1). Mean values are shown as heatmap displaying *Swap-70^-/-^* to *Swap-70^+/+^* fold-change values. The differential expression is shown as up-regulation (red) or downregulation (blue). **(J)** Representative flow cytometry of electronically gated CD4^+^Foxp3^GFP+^ Treg cells in the spleen of *Swap-70^+/+^* (blue) and *Swap-70^-/-^* (red) mice costained with CD25 and CD44, CD62L and ICOS. **(K)** Graphs depict CD25 expression levels (MFI) on electronically gated CD4^+^Foxp3^GFP+^CD25^+^Treg cells, frequency and cell numbers of CD4^+^Foxp3^GFP+^CD25^-^ Treg cells. **(L–N)** Treg cell-mediated suppression of responder T cells (Tresp) *in vitro*: FACS-purified CD4^+^CD62L^+^Foxp3^GFP-^CD25^-^ Tresp cells were co-cultured in different ratios with FACS-purified *Swap-70^+/+^* (blue) or *Swap-70^-/-^* (red) Foxp3^GFP+^ Treg cells - or as control alone (black) - in the presence of irradiated antigen presenting cells (APCs) and 1 µg/ml α-CD3ϵ. Cells were harvested and analyzed by flow cytometry at the indicated time points. **(L)** Representative flow cytometry of CD25 expression and cell proliferation dye (CPD) ef670 dilution of electronically gated Foxp3^GFP-^ Tresp cells. **(M)** Representative histogram overlays to determine CPD ef670 dilution of electronically gated Tresp cells that were co-cultured with *Swap-70^+/+^* (blue) or *Swap-70^-/-^* (red) Foxp3^GFP+^ Treg cells at a Treg : Tresp ratio of 1:1. Arrows mark proliferation dye dilution peaks (each peak represents one cell division). **(N)** Graphs depict frequencies of divided Tresp cells (left) and CD25 expression levels (MFI, right) at day 3 of culture for indicated Treg : Tresp ratios (n= 8 from 3 independent experiments). See also **Supplementary Figure S3**. *P ≤ 0.05; **P≤ 0.01; ***P ≤ 0.001; or ****P ≤ 0.0001.

To determine the function of SWAP-70 in T cells, we took advantage of the *Swap70^-/-^* x Foxp3^GFP^ mouse model, that lacks SWAP-70 (39) and carries the Foxp3^GFP^ fluorochrome reporter (38). First, fertility, mortality and cellularity of lymphoid organs were analyzed, to assess whether *SWAP-70*-deficiency affects the overall fitness of these mice, and whether there are clinical signs of systemic or organ-specific autoimmunity. For this aim, the litter sizes from homozygous *Swap-70^-/-^* mating trios (2♀ x 1♂) were analyzed and compared with their WT counterparts. The analyzes of 8 WT and 6 *Swap-70^-/-^* mating trios over a period of 15 months revealed comparable litter sizes and genotype distribution among the litters. The average litter size was 5.7 ± 2.9 pups for WT and 6.3 ± 2.9 pups for *Swap-70^-/-^* breedings. Large litters of 12-15 pups could occasionally be observed in both groups (**Figure 3A**). Evaluation of 2616 progeny from heterozygous mating trios of the *Swap-70^-/-^* x Foxp3^GFP^ strain revealed a relatively even distribution of the genotypes (**Figure 3B**): as expected, half of the descendants were heterozygous for the *Swap-70* knockout allele. However, the ratio between *Swap-70^+/+^* pups and *Swap-70^-/-^* pups was slightly biased towards the WT (26.9 vs 23.0%). The mortality rate was below 2/100 individual mice and independent of the *Swap-70* genotype (n = 2616 mice, **Figure 3C**). Organ cellularity of thymus and peripheral lymphoid organs (mesLN, scLN, spleen) was similar in *Swap-70^+/+^* and *Swap-70^-/-^* mice, implying the absence of severe multi-organ autoimmunity (**Figure 3D**). Overall, mice carrying a heterozygous or homozygous *Swap-70* knockout allele exhibited a largely healthy normal phenotype, bred like their WT counterparts and did not show obvious clinical signs of severe systemic autoimmunity.

### Impact of SWAP-70-Deficiency on Thymic and Peripheral T Cell Homeostasis

To assess the impact of SWAP-70-deficiency on T cell development and homeostasis, T cell developmental stages and populations in thymus and periphery were analyzed. Flow cytometric analysis of the CD4CD8 double negative (DN), CD4CD8 double positive (DP), CD4SP and CD8SP and Foxp3^+^ CD4SP populations revealed that frequencies and cell numbers of these compartments were similar in *Swap-70^-/-^* and *Swap-70^+/+^* mice, even though the frequency of total Foxp3^+^ CD4SP thymocytes was slightly increased in some *Swap-70^-/-^* mice (**Figure S3A**). Since SWAP-70 expression positively correlated with Helios and Nrp-1 in Treg cells in thymus and periphery (**Figure 2G** and **Supplementary Figures S2B, D**), we extended our analysis to additional Treg cell markers. *Ex vivo* analysis revealed comparable expression levels (MFI) of Foxp3^GFP^ and CD25 in the thymic Foxp3^+^ CD4SP population of *Swap-70^+/+^* and *Swap-70^-/-^* mice (data not shown), while the analysis of CD44 and CD62L provided evidence for an increased frequency of Foxp3^+^CD62L^-^CD44^+^ thymocytes in *Swap-70^-/-^* mice, accompanied by a reduction of the Foxp3^+^CD62L^+^CD44^-^ population. However, this was not reflected by absolute numbers (**Figures 3E, F** and **Supplementary Figure S3A**). In contrast, the enriched Foxp3^+^Helios^+^Nrp-1^+^ thymocyte compartment detected in *Swap-70^-/-^* mice was reflected by both, elevated frequencies and cell numbers (**Figures 3E, F** and **Supplementary Figure S3A**). Notably, expression levels of Helios were not affected, while expression of Nrp-1 was clearly increased (**Figure 3F**).

To explore whether the quantitative changes of Treg cell subsets in the thymus of *Swap-70^-/-^* mice have an impact on the Treg/Teff cell balance in adult *Swap-70^-/-^* mice, we systematically analyzed the size of the naïve and memory/effector-memory cell compartments of the CD4^+^ and CD8^+^ T cell populations in scLN, mesLN, and spleen (**Figures 3G, H** and **Supplementary Figure S3B**). Flow cytometric analysis of the total CD4 and CD8 compartments revealed comparable frequencies and cell numbers in *Swap-70^+/+^* and *Swap-70^-/-^* mice (**Supplementary Figure S3B**). Notably, the CD4^+^Foxp3^-^CD25^-^ Tcon cell compartment exhibited a slightly increased frequency of memory-type CD4^+^ T cells in the scLN of *Swap-70^-/-^* mice. However, the difference in cell numbers did not meet statistical significance (**Figure 3H** and **Supplementary Figure S3B**). Analysis of the naïve, effector-memory and central memory subsets within the CD8 compartment revealed a comparable distribution (frequencies) and population size (numbers). Albeit not being statistically significant, the CD8^+^ effector-memory compartment was somewhat increased in some *Swap-70^-/-^* mice (**Supplementary Figure S3B**). To directly assess the role of SWAP-70 in peripheral Treg cell function, we next aimed to phenotypically characterize the total Foxp3^+^ Treg cell population in the periphery. Flow cytometric analysis of the CD4^+^ T cell population revealed an increased frequency of total Foxp3^+^ Treg cells in the spleen of adult *Swap-70^-/-^* mice, which was confirmed by absolute cell numbers. Compared to their WT counterparts, the average frequency of Foxp3^+^ Treg cells increased from 9.6 to 12.3%. A similar trend could be observed in scLN (**Figures 3G, H** and **Supplementary Figure S3B**). As already indicated for CD8 and CD4 Tcon cells in the periphery, also the Treg cell population in *Swap-70^-/-^* mice shifted from naive to memory phenotype (**Figures 3G, H**, **Supplementary Figure S3B**). We expanded the flow cytometric analysis to a large panel of markers related to Treg cell activation, migration, homing and suppressor function. MFI values of electronically gated Foxp3^+^ Treg cells from *Swap-70^-/-^* mice were normalized to MFI values of electronically gated Foxp3^+^ Treg cells from age-matched *Swap-70^+/+^* littermates and **Figure 3I** summarizes the differential protein expression of the distinct Treg cell markers. The data revealed marked upregulation of cell adhesion molecules (ICAM-1, CD62L, CD103) and molecules involved in Treg cell activation and effector function (Foxp3, Helios, Nrp-1, CD38, ICOS) in *Swap-70^-/-^* mice. However, the overall expression of CD25 appeared to be downregulated *Swap-70^-/-^* Treg cells.

### Increased Frequency of Pre-Activated CD25^-^ Effector-Memory-Like Treg Cells in *Swap-70^-/-^* Mice

To address whether the shift in CD25 expression results from downregulation of CD25 surface expression or reflects an increase in the CD25^-^ Treg cell compartment, the frequency and MFI of CD25^-^, CD25^+^ and CD25^int^ Treg cell populations was analyzed. A significant increase in the frequency and number of CD25^-^ Treg cells was detected in the spleen of *Swap-70^-/-^* mice. Even though the difference did not meet statistical significance in mesLN and scLN, at least a trend of increased CD25^-^ Treg cell frequencies could be established in these organs. In *Swap-70^+/+^* mice, the average proportion of CD25^-^ Treg cells among the Foxp3^+^ population accounted for 28.2 ± 2.6% in the spleen, 20.2 ± 3.8% in the mesLN and 18.9 ± 2.6% in the scLN. In *Swap-70^-/-^* mice, the CD25^-^ compartment comprised 33.6 ± 1.2% in the spleen, 22.0 ± 2.8% in the mesLN and 21.7 ± 1.1% in the scLN. The most remarkable difference was manifested in the spleen, probably due to the overall increased presence of CD25^-^ Treg cells in this organ (**Figures 3J, K**). However, MFI values of the CD25^+^ and CD25^-^ Treg cell subset were similar in WT and KO mice in all organs analyzed (mesLN, scLN, and spleen, data not shown). In summary, these data show that the reduced CD25 MFI detected in splenic Treg cells of *Swap-70^-/-^* mice results from an enrichment of a distinct CD25^-^ Treg cell subset and is not a consequence of CD25 downregulation.

Next, we addressed whether there is a correlation between the observed decreased CD25 expression levels and increased activation marker expression in SWAP-70-deficient Treg cells. For this aim, activation marker versus CD25 expression was systematically analyzed by flow cytometry in *Swap-70^+/+^* and *Swap-70^-/-^* mice. Our data revealed that the largest part of CD44^-^Foxp3^+^ Treg cells co-expressed CD25 in both, *Swap-70^+/+^* and *Swap-70^-/-^* mice, while CD44^+^ Treg cells can acquire a CD25^+^ or CD25^-^ phenotype. Importantly, the population clearly shifted towards CD44^+^CD25^-^ in the spleen of *Swap-70^-/-^* mice. The vast majority of CD62L^+^ Treg cells displayed a CD25^+^ phenotype. CD62L^-^ Treg cells were either CD25^+^ or CD25^-^. Compared to WT, the splenic Treg cell compartment of *Swap-70^-/-^* mice comprised an increased proportion of CD62L^-^CD25^-^ cells. ICOS^+^ Treg cells were either CD25^+^ or CD25^-^, with a bias of the ICOS^+^ population towards a CD25^-^ phenotype in *Swap-70^-/-^* mice. (**Figure 3J**) Overall, these results describe an enriched CD25^-^ Treg population in the spleen of *Swap-70^-/-^* mice, co-expressing high levels of CD44, ICOS, and low levels of CD62L, which is reminiscent of antigen-experienced T cells.

### Suppressor Function of *Swap-70^-/-^* Treg Cells *In Vitro*

To determine the suppressive capacity of *Swap-70^-/-^* Treg cells, titrating numbers of FACS-purified CD4^+^Foxp3^+^ Treg cells (including CD25^+^ and CD25^-^ cells) from *Swap-70^+/+^* or *Swap-70^-/-^* mice were co-cultured with naïve polyclonal CD4^+^ Tresp cells in the presence of irradiated APCs and soluble α-CD3ϵ. As shown by upregulation of CD25 expression and cell proliferation assessed by the progressive dilution of the dye ef670, CD4^+^ Tresp cells were efficiently activated in the absence of Treg cells. At day 3 of *in vitro* stimulation, more than 90% of Tresp cells had undergone at least one cell division (**Figure 3L**, left and **N**). Co-culture with *Swap-70^+/+^* Treg cells efficiently suppressed Tresp cell activation, as revealed by the suppression of cell proliferation and expression of the early activation marker CD25 on Tresp cells in a dose-dependent manner (**Figures 3L**, middle and **M, N**). In some experiments, *Swap-70^-/-^* Treg cells appeared somewhat less potent in abrogating proliferation and upregulation of CD25 expression on Tresp cells (**Figures 3L**, right and **M, N**). The reduced ability to suppress the upregulation of CD25 was detectable as early as at day 1 of co-culture, i.e. at the time when undivided Tresp initiated TCR stimulation-induced upregulation of CD25 prior to proliferation (data not shown). At day 3, a slightly elevated frequency of divided Tresp cells, accompanied by a diminished proportion of undivided Tresp cells indicated a reduced suppressive capacity of *Swap-70^-/-^* Treg cells (**Figures 3L–M**).

### Inefficient Suppression of Osteoclastogenesis by *Swap-70^-/-^* Treg Cells *In Vitro*

**Figure 4:**
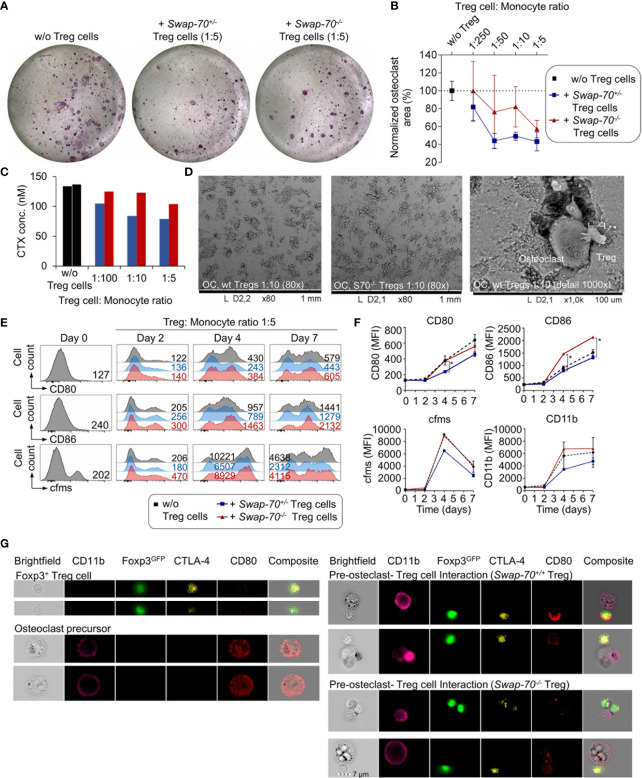
Treg-cell-mediated suppression of osteoclastogenesis *in vitro*. Bone marrow-derived macrophages (BMMs) were cultured in the absence or presence of FACS-purified *Swap-70^+/-^* or *Swap-70^-/-^* Treg cells. **(A)** Representative images of tartrate-resistant acid-phosphatase (TRAP) stained osteoclast differentiation cultures without (left) or with Treg cells (middle: *Swap-70^+/-^*, right: *Swap-70^-/-^* Treg cells) at day 6. **(B)**. Graph illustrates the impact of Treg cells on osteoclast formation assessed by the analysis of osteoclast areas (the number of TRAP-positive multinucleated cells) from co-cultures with *Swap-70^+/+^* (blue) or *Swap-70^-/-^* (red) Treg cells normalized to control cultures without Treg cells (black) (n= 3, mean values with SD shown). **(C)** Bone resorption *in vitro* assay: Pre-osteoclast-Treg cell co-culture was seeded on bone slices at different ratios, and carboxy-terminal collagen crosslinks (CTX) in the day 5 supernatant from one sample of each titration step were measured by ELISA. **(D)** The bone slices of the co-culture shown in **(C)** were harvested at day 7, dried, coated and analyzed by SEM. **(E–G)** Treg-cell-mediated downregulation of CD80/86 on pre-osteoclasts. BMMs were co-cultured with *Swap-70^+/-^* or *Swap-70^-/-^* Treg cells, harvested and analyzed by flow cytometry at the indicated time points. **(E)** Representative histogram overlays of CD80 (top), CD86 (middle) and MCSF receptor (cfms, bottom) expression on electronically gated CD4^-^Foxp3^GFP-^CD11b^+^ cells from osteoclast differentiation cultures cultured without (grey) or with Treg cells (*Swap-70^+/-^* blue; *Swap-70^-/-^* red) at indicated time points. Numbers in histograms indicate representative MFIs of gated cells. **(F)** Graphs show expression levels of CD80, CD86, cfms and CD11b (MFI) for indicated culture conditions and timepoints (one or two data points per condition from continuous co-culture). **(G)** Representative imaging flow cytometry of osteoclast-Treg cells co-culture derived cell suspensions that were analyzed for CD11b, Foxp3^GFP^, CTLA-4 and CD80 expression, showing single cell (top left: Foxp3^+^ Treg cell, bottom left: pre-osteoclast) and cluster images (top right: pre-osteoclast - *Swap-70^+/+^* Treg cell interaction, bottom right: pre-osteoclast - *Swap-70^-/-^* Treg cell interaction).

To assess the capacity of *Swap-70^-/-^* Treg cells to suppress osteoclastogenesis *in vitro*, pre-activated Foxp3^+^CD25^+^ Treg cells from *Swap-70^+/+^* or *Swap-70^-/-^* mice were co-cultured with pre-osteoclast under osteoclastogenic conditions. Consistent with previous reports (9), WT Treg cells efficiently suppressed osteoclast differentiation in a dose-dependent manner, as revealed by decreasing numbers of mature multinucleated TRAP-expressing osteoclasts cultured in the presence of increasing numbers of Treg cells. In some experiments, *Swap-70^-/-^* Treg cells appeared slightly less potent in suppressing osteoclastogenesis than SWAP-70-proficient Treg cells (**Figures 4A, B**). To determine the impact of Treg cells on the resorptive function of the osteoclasts, the co-culture was performed on bone slices using different Treg cell: monocyte ratios. In line with the observed reduced capacity of *Swap-70^-/-^* Treg cells to suppress osteoclastogenesis, the analysis of the bone resorption marker CTX revealed moderately elevated CTX levels in the supernatants of co-cultures of osteoclast precursors and *Swap-70^-/-^* Treg cells compared to co-cultures with *Swap-70^+/+^* Treg cells in all ratios analyzed (**Figure 4C**). Although the observed trend was statistically not significant, and the CTX assay replicates are reflected by the titration steps, the diminished ability to suppress osteoclast differentiation and function is reminiscent of and in line with the moderate differences we observed in standard suppression assays (**Figures 3L–N**). Overall, we soughed to address our question with multiple approaches, and found that different analyses converged on the same result, proposing that *Swap-70*^-/-^ Treg cells are compromised with regard to their suppressor function. Representative SEM images of Treg cell and osteoclast interactions on bone slices demonstrated direct cell-cell interactions of giant bone-resorptive mature osteoclasts with the much smaller Treg cells (**Figure 4D** right). Importantly, we found less cell clusters in co-cultures of osteclasts with *Swap-70^-/-^* Treg cells (**Figure 4D** middle) than with *Swap-70^+/+^* Treg cells (**Figure 4D** left).

### Inefficient *Swap-70^-/-^* Treg Cell-Mediated Downregulation of CD80/CD86 on Osteoclast Precursor Cells

Since SWAP-70 has been shown to be required for endocytosis in DCs (27), we assessed whether *Swap-70^-/-^* Treg cells are capable of efficient CD80 and CD86 down-modulation on osteoclast precursors. Flow cytometric analysis revealed progressive upregulation of CD80, CD86, the M-CSF receptor cfms, and CD11b during osteoclast maturation *in vitro*. Co-culture of osteoclast progenitors and SWAP-70-proficient Treg cells resulted in diminished upregulation of CD80 (day 7: 70 ± 3%, compared to culture without Treg cells) and CD86 (day 7: 87 ± 2%), with a more efficient down-modulation of CD80, and a concomitant limited expression of cfms (day 7: 65 ± 6%) and CD11b (day 7: 81 ± 16%) (**Figures 4E, F**). In contrast, *Swap-70^-/-^* Treg cells failed to efficiently downregulate CD80 (day 7: 86 ± 3%) and CD86 (day 7: 140 ± 7%), which is significantly different from *Swap-70^+/+^* Treg cells and moreover had no impact on cfms (day 7: 108 ± 18%) and CD11b (day 7: 119 ± 33%) expression during osteoclastogenesis. These observations are in line with the reduced suppressive capacity of *Swap-70^-/-^* Treg we described above (**Figures 3L**–**N** and **4A**–**C**).

To determine whether *Swap-70^-/-^* Treg cells are able to interact directly with osteoclast precursors, osteoclast-Treg cell co-culture were analyzed at day 2 by imaging flow cytometry, allowing the visualization of cell-cell interactions and subcellular protein localization. As we have shown previously (9), imaging analysis of CD11b/Foxp3^GFP^-gated cell clusters indicated physical interaction of Foxp3^+^CTLA4^+^ Treg cells with CD11b^+^ osteoclast precursor cells. CD11b^+^ cells that were detected in co-existence with Foxp3^+^ Treg cells displayed an overall reduced surface expression of CD80, as compared to CD11b^+^ single cells, while CD80 expression was clearly detectable in Foxp3^+^CTLA4^+^ Treg cells, suggesting CD80 uptake by direct interaction between the two cell types and trans-endocytosis. (**Figure 4G**). However, we detected no difference between Foxp3^GFP+^*Swap-70^+/+^* and Foxp3^GFP+^*Swap-70^-/-^* Treg cells with regard to the up-take of CD80, proposing that trans-endocytosis is not, or only moderately compromised in Treg cells that lack SWAP-70.

## Discussion

Dynamic rearrangements of the actin cytoskeleton in antigen-specific T cells represent a prerequisite for the effective polarization, migration and exertion of specific functions such as the formation of an immunological synapse. Since SWAP-70 modulates F-actin rearrangements, regulates integrin activity, and thus cell migration and adhesion in a variety of cells of hematopoietic origin (26, 28–31, 33, 35), it seems tempting to speculate that the selective constitutive expression of SWAP-70 in the Foxp3^+^ Treg cell subset of the CD4^+^ T cell lineage (37) might represent a direct consequence of Foxp3-dependent transcriptional regulation and that SWAP-70 participates in the regulation of actin dynamics in Treg cells. Here, we follow up on this initial observation and perform detailed studies on the role of SWAP-70 in Foxp3^+^ Treg cell biology, identifying SWAP-70 as an important regulator of Treg cell homeostasis and suppressor function involved the interaction of Treg cells with osteoclasts. We found that *Swap-70* is constitutively expressed in Foxp3^+^ Treg cells, which is in line with initial studies (37), and that *Swap-70* expression is upregulated upon TCR-mediated stimulation in initially naïve *Swap-70^-^* CD4^+^ Tcon. We further established that *Swap-70* mRNA expression is under the direct control of Foxp3/NF-AT. These findings are consistent with the notion that Foxp3 controls Treg cell function through cooperation with NF-AT (52) and propose *Swap-70* as a member of the canonical Treg cell signature (37, 44).

When we analyzed the spatial localization of SWAP-70 upon TCR engagement, we observed that cytosolic SWAP-70 is recruited to the immunological synapse (IS) exclusively in Foxp3^+^ Treg cells, which was in contrast to CD4^+^ Tcon cells. The localization of SWAP-70 at the Treg-APC interface provides first evidence for an F-actin-dependent function of SWAP-70 selectively in primary Treg cells and suggest that SWAP-70 has a critical role in the formation of a stable IS between Treg cells and APCs. In contrast, SWAP-70`s only homolog DEF6 is constitutively expressed and involved in TCR-mediated cytoskeletal dynamics and recruitment in CD4^+^ Tcon cells (53, 54), emphasizing the distinct function of SWAP-70 in Treg cells.

The overall mature Foxp3^+^ Treg cell compartment has been shown to exhibit a remarkable phenotypic heterogeneity and comprises several subsets, as defined by their developmental origin, anatomical location, activation status and functional properties. The differential transgenic expression of GFP in RFP^+^ Treg cells of Foxp3^RFP/GFP^ mice has been shown to faithfully discriminate tTreg and pTreg cells (40, 47). At least in some experimental settings, this can also be achieved by the expression analysis of the Ikaros transcription factor Helios (Ikzf2) (51) and the surface marker Nrp-1 (55, 56), but endogenous (i.e. non-transgenic) markers to unambiguously discriminate tTreg and pTreg cell subsets have yet to be defined (57). The concomitant induction of SWAP-70 expression with CD25, Helios and Nrp-1 in the instructive phase of tTreg cell lineage commitment (stage I) and progressive up-regulation to high levels during developmental progression and tTreg lineage consolidation (stage II and III) suggests a mechanism of TCR-mediated induction and subsequent Foxp3-mediated amplification/stabilization of SWAP-70 expression during tTreg cell development and supports the assumption that SWAP-70 is a Foxp3 target gene. Together with the observed correlation of SWAP-70 expression with the expression of CD44, Helios and Nrp-1 in mature Treg cells, our data suggest that - similar to Helios and Nrp-1 - SWAP-70 cannot be considered as a tTreg cell lineage marker, but rather propose SWAP-70 to be a putative marker for Helios^hi^Nrp-1^hi^Treg cells. In summary, the results demonstrate that SWAP-70 is differentially expressed during Foxp3^+^ Treg cell development and activation (**Supplementary Figure S2E**).

A role of SWAP-70 in T cells during thymic development and peripheral homeostasis is supported by the marked upregulation of cell adhesion molecules and molecules involved in their activation and effector function (ICAM-1, CD103 and Helios, Nrp-1, CD38, ICOS), in conjunction with increased proportions of effector/memory-type CD4^+^ and CD8^+^ T cells and an enriched CD25^-^ Treg population in the spleen of *Swap-70^-/-^* mice. *Swap-70^-/-^* CD25^-^ Treg co-express high levels of CD44, ICOS and low levels of CD62L, which is reminiscent of activation-experienced Treg cells (58).

Increased numbers of Foxp3^+^CD25^low^CD4^+^ Treg cells were detected in peripheral blood of patients with autoimmune diseases such as systemic lupus erythematosus, multiple sclerosis, type 1 diabetes and rheumatoid arthritis (59–63), and it was suggested that this may reflect an attempt to regulate an overt autoimmune response of pathogenic T cells and might contribute to the perpetuation of chronic inflammation (61, 64). Furthermore, it has recently been reported that mice with diminished CD25 expression harbor decreased numbers of CD62L^+^CD44^−^ Treg cells indicating an impact of impaired IL-2R signaling on Treg cell stability (65). Efficient IL-2/IL-2R signaling is essential for Treg cell homeostasis and function, and competition for IL-2 has been suggested as one basic mechanism of immune suppression by Treg cells (66, 67). When we analyzed the *in vitro* function of *Swap-70^-/-^* Treg cells, we found that the suppression of proliferation and upregulation of CD25 expression on Tresp cells was somewhat less effective compared to SWAP-70*-*proficient Treg cells. As the reduced suppression of CD25 upregulation on Tresp cells was already apparent at day 1 of co-culture, we hypothesize that *Swap-70^-/-^* Treg cells suppress less effective due to their - at least initially - lower IL-2R expression and then upregulate IL-2R through local IL-2 secretion by Tresp and consequently could suppress more efficient.

**Figure 5:**
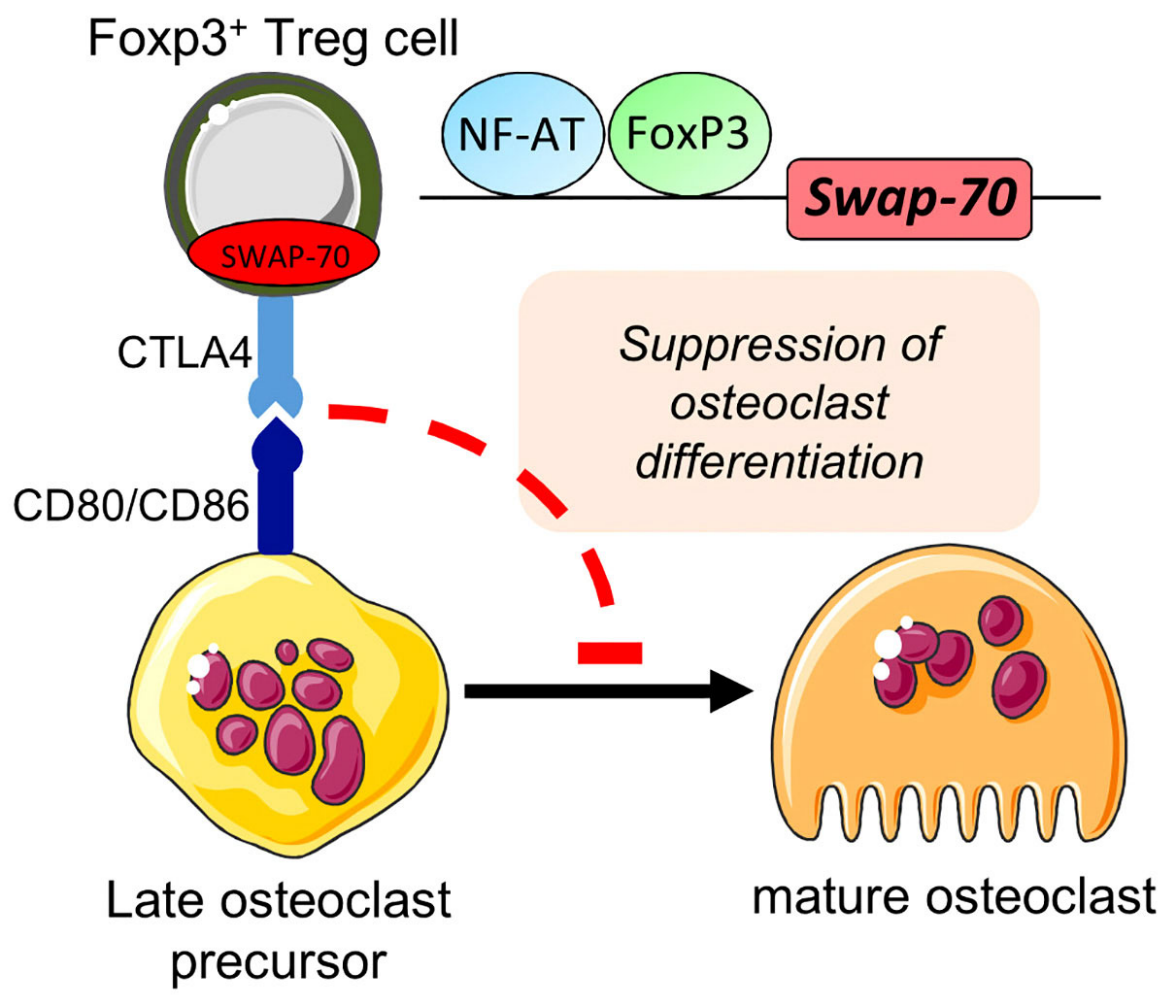
Proposed role for SWAP-70 in the direct interplay between Foxp3^+^Treg cells and osteoclast precursors. Bone homeostasis mediated through Foxp3^+^ Treg cells (*via* CTLA-4 interacting with CD80/CD86) underlies SWAP-70-dependent mechanisms. Adapted from 9. This figure was in part created with modified Servier Medical Art templates, licensed under a Creative Commons Attribution 3.0 Unported license: http://smart.servier.com.

To elucidate the role of SWAP-70 in osteoimmunology and in particular the interplay of Treg cells and osteoclasts, we performed co-cultures of Treg cells and osteoclast precursor cells. These experiments revealed an inefficient suppression of osteoclastogenesis and resorptive function by *Swap-70^-/-^* Treg cells *in vitro* correlating with diminished Treg cell-adhesion to osteoclasts and most importantly that *Swap-70^-/-^* Treg cells failed to efficiently downregulate CD80 and CD86 expression during osteoclastogenesis (**Figure 5**). Although *Swap-70^-/-^* Treg cells trans-endocytosed CD80 as efficiently as SWAP-70*-*proficient Treg cells, these findings suggest that SWAP-70 might represent a novel link between the immune and the skeletal system. Considering previously reported roles of SWAP-70 in integrin-mediated adhesion, we suggest that SWAP-70 is required for efficient Treg cell-mediated regulation of osteoclastogenesis through regulating integrin-mediated adhesion and cytoskeletal re-arrangements.

Together, our analysis of the suppressive capacity of *Swap-70^-/-^* Treg cells indicate a general but modest defect in Treg suppressor function in *Swap-70^-/-^* mice. However, *Swap-70^-/-^* mice did not show obvious clinical signs of a severe systemic autoimmune disease. Since we found *Swap-70* to be expressed in activated Teff cells, our data suggest a scenario in which the function of SWAP-70 in Teff is crucial for the establishment of autoimmunity in the context of SWAP-70-deficient Treg cells. On the other hand, it has been described that mice deficient in both SWAP-70 and its homolog DEF6 develop systemic lupus erythematosus (68), indicating that DEF6 expression might at least partially compensate for defects in *Swap-70^-/-^* mice. It is a limitation of our present study that we did not address this hypothesis, and it will be important to determine in future studies how these two proteins control and balance Treg cell function in the context of osteoimmunology.

In summary, our study uncovers a previously unrecognized role of the F-actin binding protein SWAP-70. We propose Swap-70 as a member of the so called canonical Treg cell signature and provide first evidence for a crucial role of SWAP-70 in Treg cell homeostasis and suppressor function. Collectively, our data define a novel role of SWAP-70 in osteoimmunology and may thus have future implications for studies on bone disorders associated with functional defects of Treg cells, including rheumatoid arthritis.

## Data Availability Statement

The original contributions presented in the study are included in the article/**Supplementary Material**. Further inquiries can be directed to the corresponding author.

## Ethics Statement

The animal study was reviewed and approved by Regierungspräsidium Dresden.

## Author Contributions

SD designed, conducted, and analyzed the experiments, contributed to data interpretation and manuscript writing. SM and AS performed experiments. TK analyzed experiments and contributed to data interpretation. AG, RJ, and KK conceived the research. AG and KK guided its design, analysis and interpretation, and wrote the manuscript. All authors contributed to the article and approved the submitted version.

## Funding

AG was supported by a DFG grant (GA1576/1-2) within the SPP1468 IMMUNOBONE and two intramural grants from the Technische Universität Dresden (MeDDrive Program of the Medical Faculty and Maria Reiche Program). In addition, AG and KK received funds from the FZT 111 (DFG, Center for Regenerative Therapies Dresden, Cluster of Excellence).

## Conflict of Interest

The authors declare that the research was conducted in the absence of any commercial or financial relationships that could be construed as a potential conflict of interest.

## Publisher’s Note

All claims expressed in this article are solely those of the authors and do not necessarily represent those of their affiliated organizations, or those of the publisher, the editors and the reviewers. Any product that may be evaluated in this article, or claim that may be made by its manufacturer, is not guaranteed or endorsed by the publisher.

## Supplementary Material

Supplementary Figure 1 SWAP-70 is expressed in CD4^+^Foxp3^+^ Treg cells. Flow cytometric analysis of SWAP-70 expression in indicated T cell subsets. Representative dot plots with gating strategy (arrows) and representative histograms of intracellularly (IC) stained SWAP-70 of indicated populations in thymus (left) and periphery (scLN, right). SWAP-70 expression in naïve (CD62L^+^CD44^-^) and memory-type (CD62L^-^CD44^+^) CD4^+^Foxp3^+^ Treg cells is shown at the bottom right. Numbers in dot plots and representative histograms indicate representative frequencies and MFIs of gated cells within the respective gates, respectively. Data are representative of 4 independent experiments.

Supplementary Figure 2 FACS-purification of CD4SP CD25^+^ Treg precursor stages according to the differential expression of Foxp3^RFP^ and Foxp3^GFP^ and co-expression of SWAP-70, Helios and Nrp-1. **(A)** Representative dot plots of presort gating strategy with (bottom) and without (top) CD25-bead enrichment. Lines with arrowheads in dot plots illustrate the gating strategy. **(B)** Representative flow cytometric analysis of FACS-purified Foxp3^RFP-^Foxp3^GFP-^ CD25^+^ CD4SP (stage I), Foxp3^RFP+^Foxp3^GFP-^ CD25^+^ CD4SP (stage II) and Foxp3^RFP+^Foxp3^GFP+^CD25^hi^ CD4SP (stage III) thymocytes for IC Helios, Nrp-1 and IC SWAP-70 co-expression. **(C)** Representative dot plots of presort gating strategy with (bottom) and without (top) CD25-bead enrichment for pTreg and tTreg sorts (scLN shown). **(D)** Representative flow cytometric analysis of FACS-purified pTreg (Foxp3^RFP+^Foxp3^GFP-^ CD25^+^CD4^+^) and tTreg cells (Foxp3^RFP+^Foxp3^GFP+^ CD25^+^CD4^+^) for IC Helios, Nrp-1 and IC SWAP-70 co-expression. Numbers in representative dot plots indicate frequencies of the gated population. **(E)** Schematic summary of SWAP-70 protein expression during Treg cell development. SWAP-70 expression pattern based on flow cytometric analysis of thymocytes from Foxp3^RFP/GFP^ mice and extrathymic Foxp3^+^ Treg cells from Foxp3^GFP^ x *Swap70^+/+^* mice. SWAP-70 expression was already detectable in the instructive phase of Treg cell lineage commitment (stage I) in the thymus and was highly expressed during consolidation phases (stage II and III), concomitant with Foxp3, indicating a TCR mediated induction of SWAP-70 *via* NF-AT. Constitutive expression of SWAP-70 in Treg cells in the periphery is stabilized by Foxp3.

Supplementary Figure 3 Impact of SWAP-70 on immune homeostasis. **(A)** Graphs depict frequencies and cell numbers of the indicated populations in the thymus of *Swap-70^+/+^* (blue) and *Swap-70^-/-^* (red) x Foxp3^GFP^ mice. **(B)** Frequencies and cell numbers of the indicated populations in scLN, mesLN and spleen of *Swap-70^+/+^* (blue) and *Swap-70^-/-^* (red) x Foxp3^GFP^ mice. Symbols and horizontal lines indicate individual mice and mean values, respectively (circles: 8-9-week-old; triangles: 10-13-week-old mice; n= 6 per genotype).
