# Supplementary figures and images for: Role of Dynamic Actin Cytoskeleton Remodeling in Foxp3+ Regulatory T Cell Development and Function: Implications for Osteoclastogenesis
Source: Front Immunol. 2022 Mar 11;13:836646. doi: 10.3389/fimmu.2022.836646 (PMC8963504; doi:10.3389/fimmu.2022.836646)

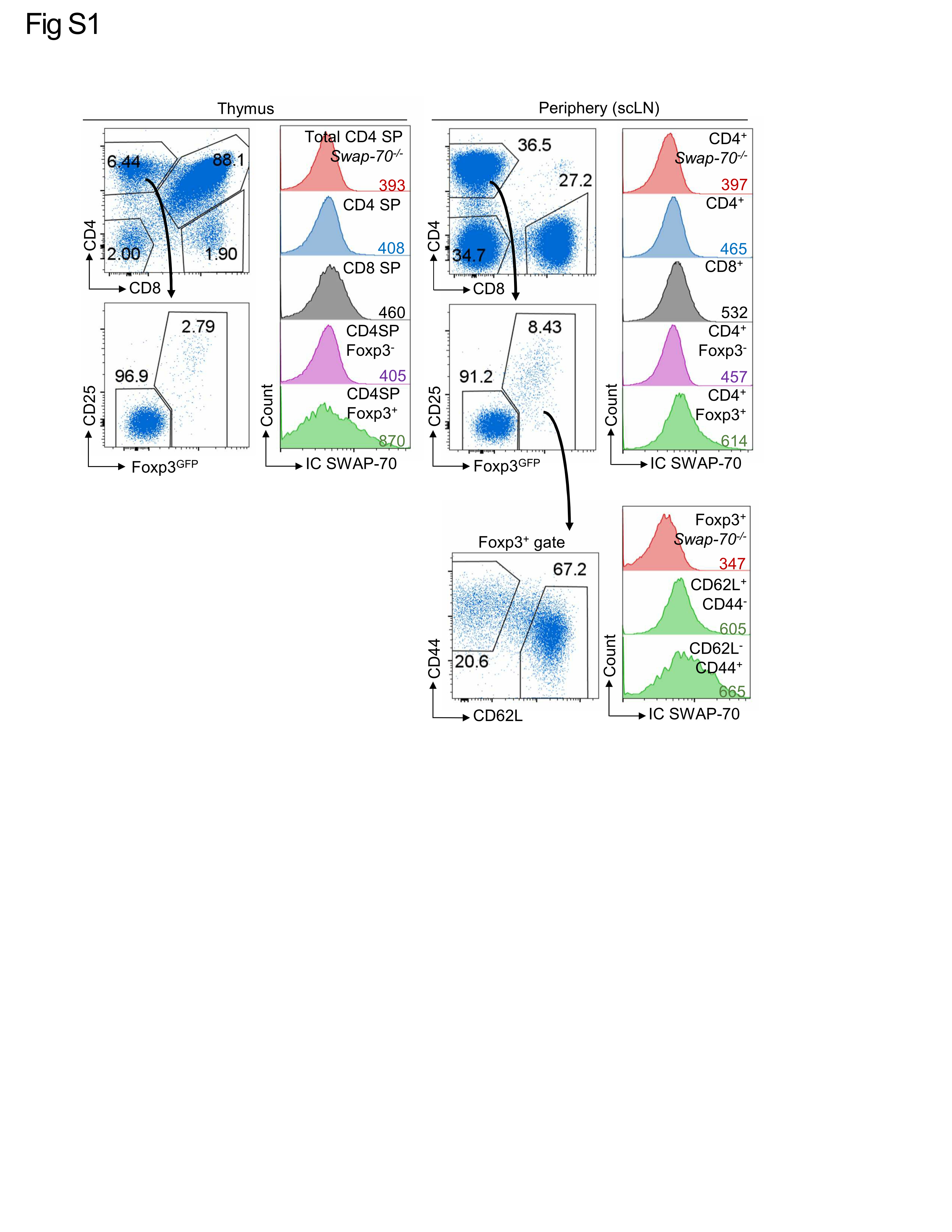

Supplement: Supplementary Figure 1 — SWAP-70 is expressed in CD4+Foxp3+ Treg cells. Flow cytometric analysis of SWAP-70 expression in indicated T cell subsets. Representative dot plots with gating strategy (arrows) and representative histograms of intracellularly (IC) stained SWAP-70 of indicated populations in thymus (left) and periphery (scLN, right). SWAP-70 expression in naïve (CD62L+CD44-) and memory-type (CD62L-CD44+) CD4+Foxp3+ Treg cells is shown at the bottom right. Numbers in dot plots and representative histograms indicate representative frequencies and MFIs of gated cells within the respective gates, respectively. Data are representative of 4 independent experiments. [file Image_1.tif]

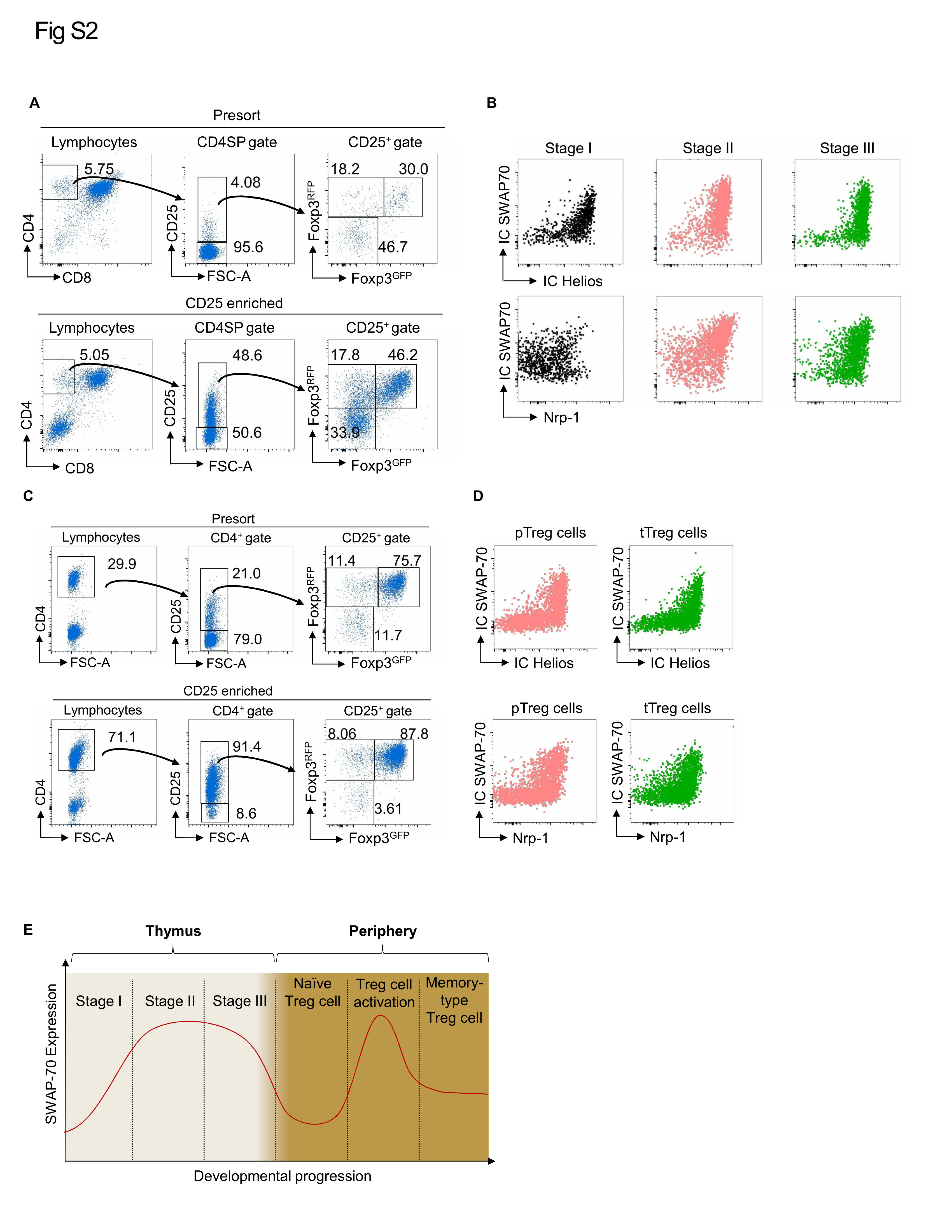

Supplement: Supplementary Figure 2 — FACS-purification of CD4SP CD25+ Treg precursor stages according to the differential expression of Foxp3RFP and Foxp3GFP and co-expression of SWAP-70, Helios and Nrp-1. (A) Representative dot plots of presort gating strategy with (bottom) and without (top) CD25-bead enrichment. Lines with arrowheads in dot plots illustrate the gating strategy. (B) Representative flow cytometric analysis of FACS-purified Foxp3RFP-Foxp3GFP- CD25+ CD4SP (stage I), Foxp3RFP+Foxp3GFP- CD25+ CD4SP (stage II) and Foxp3RFP+Foxp3GFP+CD25hi CD4SP (stage III) thymocytes for IC Helios, Nrp-1 and IC SWAP-70 co-expression. (C) Representative dot plots of presort gating strategy with (bottom) and without (top) CD25-bead enrichment for pTreg and tTreg sorts (scLN shown). (D) Representative flow cytometric analysis of FACS-purified pTreg (Foxp3RFP+Foxp3GFP- CD25+CD4+) and tTreg cells (Foxp3RFP+Foxp3GFP+ CD25+CD4+) for IC Helios, Nrp-1 and IC SWAP-70 co-expression. Numbers in representative dot plots indicate frequencies of the gated population. (E) Schematic summary of SWAP-70 protein expression during Treg cell development. SWAP-70 expression pattern based on flow cytometric analysis of thymocytes from Foxp3RFP/GFP mice and extrathymic Foxp3+ Treg cells from Foxp3GFP x Swap70+/+ mice. SWAP-70 expression was already detectable in the instructive phase of Treg cell lineage commitment (stage I) in the thymus and was highly expressed during consolidation phases (stage II and III), concomitant with Foxp3, indicating a TCR mediated induction of SWAP-70 via NF-AT. Constitutive expression of SWAP-70 in Treg cells in the periphery is stabilized by Foxp3. [file Image_2.tif]

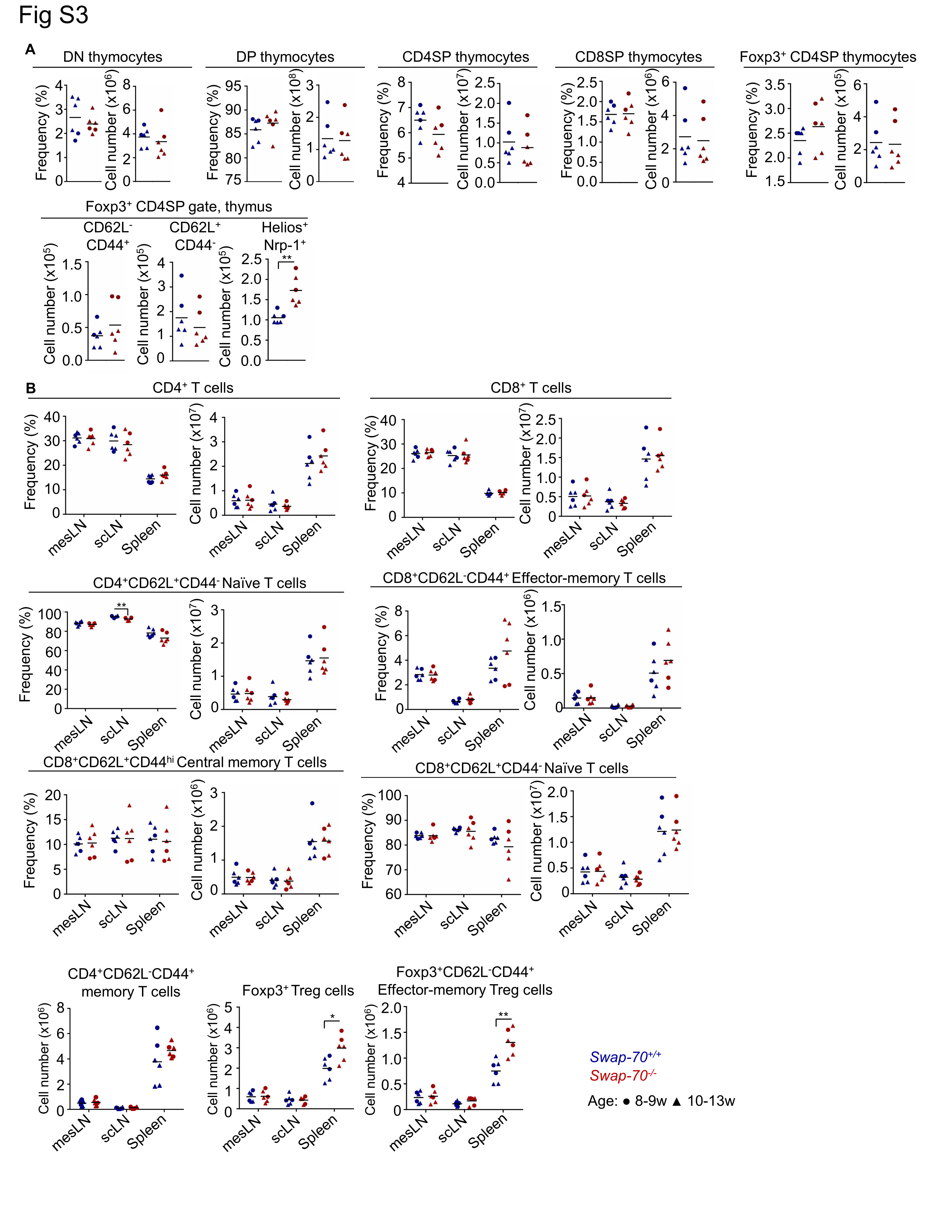

Supplement: Supplementary Figure 3 — Impact of SWAP-70 on immune homeostasis. (A) Graphs depict frequencies and cell numbers of the indicated populations in the thymus of Swap-70+/+ (blue) and Swap-70-/- (red) x Foxp3GFP mice. (B) Frequencies and cell numbers of the indicated populations in scLN, mesLN and spleen of Swap-70+/+ (blue) and Swap-70-/- (red) x Foxp3GFP mice. Symbols and horizontal lines indicate individual mice and mean values, respectively (circles: 8-9-week-old; triangles: 10-13-week-old mice; n= 6 per genotype). [file Image_3.tif]
